# Evaluation of the In Vitro Wound-Healing Potential of Ayahuasca

**DOI:** 10.3390/molecules27185760

**Published:** 2022-09-06

**Authors:** Joana Gonçalves, Ângelo Luís, Eugenia Gallardo, Ana Paula Duarte

**Affiliations:** 1 Centro de Investigação em Ciências da Saúde (CICS-UBI), Universidade da Beira Interior, Av. Infante D. Henrique, 6200-506 Covilhã, Portugal; 2 Laboratório de Fármaco-Toxicologia, UBIMedical, Universidade da Beira Interior, Estrada Municipal 506, 6200-284 Covilhã, Portugal

**Keywords:** ayahuasca, wound-healing activity, PAMPA assay, HPLC-DAD

## Abstract

Ayahuasca is an Amazonian drink, which contains β-carboline alkaloids and *N*,*N*-dimethyltryptamine. The aim of this study was to evaluate the healing potential of decoctions of a commercial mixture, four individual plants and four mixtures of two plants used in the ayahuasca preparation. Thus, the cytotoxic potential of the samples was evaluated and a wound-healing assay was performed with a NHDF cell line. Subsequently, a parallel artificial membrane permeability assay was also performed, to verify if any psychoactive compound could be absorbed by skin fibroblasts. The integrity and permeability of the cell layer were also evaluated, using the transepithelial electrical resistance assay and Lucifer yellow permeability assay, respectively. The compounds absorbed by the cell layer were quantified by high-performance liquid chromatography coupled to a diode array detector. The results showed that only one sample showed cytotoxicity and all the others promoted the migration of skin fibroblasts. Additionally, it was also verified that β-carbolynic alkaloids and *N*,*N*-dimethyltriptamine were not absorbed by the cell layer, and in general, did not interfere with its permeability and integrity. To the best of our knowledge, this is the first study where ayahuasca’s wound-healing potential was evaluated.

## 1. Introduction

Ayahuasca is a psychoactive beverage, originally prepared from the stems of *Banisteriopsis caapi* (*B. caapi*) and leaves of *Psychotria viridis* (*P. viridis*), although there are currently other variations [1,2]. Also known as *Hoasca*, *Caapi*, *Yajé/Yagé*, or *Daime*, among other names, it may have a variable composition, and more than one hundred different plants used in its preparation have been documented [1,3]. The active compounds present in this beverage are *N*,*N*-dimethyltryptamine (DMT) from *P. viridis* and β-carbolines from *B. caapi* [4,5]. DMT is a tryptamine with psychoactive action, which acts as an agonist of serotonergic receptors 2A (5HT2AR) and non-opioid intracellular receptor sigma 1 (SIGMAR1), exerting effects comparable to those of psilocybin, mescaline or LSD [6]. This psychoactive compound is not bioavailable when ingested orally, since it is rapidly metabolised by peripheral monoaminoxidase-A (MAO-A) [6]. However, β-carbolinic alkaloids, namely harmine, harmaline and tetrahydroharmine (THH), have inhibiting effects on MAO-A, so the co-administration of these compounds with DMT prevents their degradation [1,7]. Thus, the psychoactive compound accesses the bloodstream and later the central nervous system, exerting its psychoactive effects [8]. Additionally, THH acts as an inhibitor of serotonin reuptake, enhancing the effects of DMT [9].

Ayahuasca users describe effects as changes in hearing, visual sensations, space and time awareness or even emotional and cognitive changes [1,4,10]. Spiritual experiences, such as connections with mythical or religious entities, are also often reported [4]. From the physical point of view, it is common to experience effects such as nausea, diarrhea or vomiting [6]. Ayahuasca has been used traditionally for over a thousand years for medicinal and spiritual purposes by indigenous peoples of the Amazon [4,11]. However, in recent decades, several studies have pointed to psychological benefits, namely in the treatment of depression, anxiety, drug dependence or obsessive-compulsive disorder [8,12,13,14,15]. Other more subtle effects, such as improvement in assertiveness, trust, optimism, maturity, or decreased neuroticism, have also been associated with ayahuasca consumption [13,16,17]. Recently, the anti-inflammatory and antimicrobial activities of ayahuasca extracts have also been verified [2].

Ayahuasca consumption has been increasing in recent decades all over the world [7]. Despite all the beneficial effects reported, and there is evidence that the consumption of this substance in a controlled environment is not associated with such consequences as psychotic outbreaks, this increase in demand for the decoction has led to questions about the possible negative effects associated with its consumption; however, it has also led to interest in its potential therapeutic effects [2].

The particular case of wounds represents a major challenge for health, as there is a high resource expenditure involved in trying to improve people’s well-being [18]. Thus, several studies have been carried out in an attempt to find compounds of natural origin that allow wound healing [18,19,20]. Goulart da Silva et al. [21] describe in their review the potential anti-inflammatory properties of ayahuasca and its implications in neurological and psychiatric diseases. The authors mention that this is probably linked to its anti-neuroinflammatory action, mainly attributed to dimethyltryptamines (*N*,*N*-dimethyltryptamine and 5-methoxy-*N*,*N*-dimethyltryptamine), which act as systemic regulators of inflammation and immune homeostasis, but also to sigma-1 receptors. The study of Dakic et al. [22] corroborates this potential. The authors suggest that the compound modulates anti-neuroinflammatory response through the nuclear factor of activated T cells and nuclear factor kappa B pathways, which are downregulated through the toll-like receptor and G protein-coupled receptors. These anti-inflammatory effects are consistent with reported results in which the inflammatory release of cytokines and chemokines was blocked [21].

Additionally, the antioxidant activity of ayahuasca was documented in previous studies, as well as the presence of phenolic compounds and flavonoids, which are associated with this activity, since they promote the scavenging of free radicals [2,18].

Furthermore, its anti-inflammatory and antimicrobial properties suggest the potential of ayahuasca in wound healing.

In this context, this work aimed to evaluate in vitro the healing activity of four individual plants, a commercial mixture and four plants generally used in the preparation of ayahuasca decoctions, using normal human dermal fibroblasts (NHDF) through a scraping test. The potential cytotoxicity of the extracts and the eventual skin absorption of the psychoactive compounds were also evaluated by 3-[4,5-dimethylthiazol-2-yl]-2,5-diphenyltezolium bromide (MTT) and parallel membrane permeability assay (PAMPA), respectively.

## 2. Results and Discussion

The demand for natural products with potential bioactive properties has increased. In previous works, ayahuasca has been shown to have some therapeutic benefits, namely antioxidant, antimicrobial and anti-inflammatory properties [2]. Thus, in this work, one decoction was prepared from a commercial mixture, four from individual plants used in the preparation of ayahuasca, and four from a mixture of plants (with two different plant materials, one a source of β-carboline alkaloids and the other a source of DMT). Wound-healing potential was assessed in all nine samples.

### 2.1. Evaluation of Cell Viability

The cytotoxicity of all samples was evaluated at concentrations of 250 and 500 mg/L. The MTT assay was used for this purpose. This is a method employed used in the detection of cellular alterations at the metabolic level [23]. This assay consists of the conversion, by action of a mitochondrial reductase, of the yellow dye [3-(4,5-dimethylthiazol-2-yl)-2,5-diphenyltetrazolium bromide] into the purple dye formazan [9,23]. After analysing the results, it was found that the *P. harmala* sample, at both concentrations, showed a high decrease in cell viability, so this sample was eliminated in the subsequent phase of this study (Table 1). All other samples showed no decrease in cell viability. To our knowledge, no studies have evaluated the cytotoxicity of ayahuasca samples on human skin fibroblasts, so it is not possible to establish a comparison. However, in a study that evaluated the cytotoxicity of *B. caapi* extracts in six cell lines, no cytotoxicity was observed [24]. In another study by Katchborian-Neto et al. [25], where the cytotoxicity of ayahuasca samples in SH-SY5Y cells was evaluated, it was found that there was no decrease in cell viability. On the other hand, studies where cancer cells were exposed to ayahuasca samples showed a decrease in cell viability [26,27].

### 2.2. Evaluation of the In Vitro Wound-Healing Activity

Natural products have an important capacity in the reconstruction of skin lesions, as they lead to the proliferation of fibroblasts. Plant extracts have been reported to contain cell adhesion molecules, growth factors, and signalling molecules, which aid in the regeneration process and consequent wound healing [28].

In the present study, the healing potential of 8 decoctions used in the preparation of ayahuasca was evaluated, using the wound scratch test. The evolution of the scratch created was monitored using microscopic images (Table 2) and the distance between the margins of the lesion was calculated (Table 3). Analysing the images that showed the evolution of the distance between the margins of the lesion and comparing them with the control samples, it was possible to verify that, in general, all the samples showed a great decrease in the lesion, and after 24 h of incubation, the samples of *M. hostilis* + *P. harmala* at 500 mg/L and *P. viridis* + *B. caapi* at 250 mg/L showed the best results. However, analysing the evolution of the distance calculated, only the sample of the commercial mixture at 250 mg/L after 2 h of incubation did not show a significant decrease. All other samples at different evaluation times, as well as the commercial mixture at 250 mg/L at the other times (8 h, 12 h and 24 h) showed a significant decrease in lesion margins compared to the control.

These results are indicative of the healing activity of the samples tested. It is possible to observe in the images of Table 2 the migration of the fibroblasts incubated with the samples at different concentrations. In these images, it was possible to verify that the lesions closed over time, which was in accordance with the distance calculated between the margins of the lesion. As far as we know, to date there are no studies where the healing potential of ayahuasca has been evaluated, and it is not possible to compare the results now obtained. However, these results can be explained by the antioxidant and anti-inflammatory activity previously studied in these samples [2], since it is reported that antioxidant activity and healing properties coexist in plant extracts [28]. Wound healing consists of the reconstruction of the lesion, involving several interactions between epithelial cells, growth factors, cytokines and chemokines. It has been reported that natural products, namely plant extracts, are involved in the proliferation of fibroblasts and keratinocytes, and may contain cell adhesion molecules, growth factors and cell signalling molecules, which can promote lesion reconstruction [28]. This in vitro assay, which, unlike conventional assays used to determine the healing properties of plant material, was non-invasive, allowed the screening of several samples with antibacterial, anti-inflammatory and antioxidant properties, which are important in wound healing [2,20].

Ayahuasca has psychoactive compounds in its constitution, and since the samples showed healing potential, we evaluated whether compounds such as DMT, harmine, harmaline, harmol, harmalol and THH can cross the NHDF cell layer, being absorbed and becoming accessible to the bloodstream. For this, a PAMPA assay was performed, and parameters such as permeability and cell layer integrity were also evaluated.

### 2.3. Evaluation of the Electrical Resistance of the Cell Transendothelial Membrane

The integrity of the cell layer was assessed using the TEER assay, the results being shown in Table 4. This assay, which is able to assess changes in intercellular junctions and monitor the integrity of the cell monolayer, was performed before and after incubation with the extracts [29]. After incubation with the extracts, a new TEER measurement was performed, where it was possible to verify that during cell incubation with the sample *P. viridis* + *P. harmala* at 500 mg/L there were significant changes in the integrity of the cell monolayer. In all other samples there was no significant change in cell layer integrity. To date, there are no studies with ayahuasca samples where the TEER assay was performed on NHDF cells. However, in another study carried out by our research group, where the TEER assay was performed in intestinal adenocarcinoma cells, after incubation with ayahuasca samples, no significant changes were observed in the integrity of the cells, however the concentrations of the samples used were much lower [9].

### 2.4. Evaluation of Cell Monolayer Permeability

Cell layer permeability was evaluated by the Lucifer yellow permeability assay, the results being shown in Table 5. This assay was used to assess the permeability of a cell monolayer by measuring the passive diffusion of specific molecules, as is the case with Lucifer yellow [30]. Analysing the results, it was possible to verify that, compared to the control, significant changes only occurred when the cells were incubated with the *P. viridis* + *P. harmala* sample at 500 mg/L. Studies suggest that permeability and TEER measurement are inversely related; that is, a decrease in TEER values is accompanied by an increase in permeability [31,32]. Comparing the results obtained in this assay with the results obtained in the TEER assay, we verified that they were in agreement. These results suggest that, with the exception of the sample of *P. viridis* + *P. harmala* at 500 mg/L, there were no changes in cell barrier function or intracellular spaces, and consequently, in membrane permeability [31,32]. So far, there are no studies with samples of ayahuasca where this assay has been carried out in NHDF cells, so it is not possible to establish a comparison. However, as previously mentioned, our research group carried out a study in which the Lucifer yellow permeability assay was performed on intestinal adenocarcinoma cells, and after incubation with ayahuasca samples, no significant changes were observed in the permeability of the cells [9]. However, it should be noted that the concentrations of the samples used were much lower [9].

### 2.5. Characterisation of the Main Compounds after Cell Incubation

After performing the PAMPA assay, aliquots were collected from the basolateral part of each well and the amounts of the compounds present in the aliquots were quantified by HPLC-DAD. This analytical method, previously developed and validated, proved to be linear between 0.16 and 10.00 µg/mL for harmol, THH, harmaline and harmine; between 0.31 and 10.00 µg/mL for harmalol and between 0.031 and 1.00 µg/mL for DMT, with coefficients of determination higher than 0.997. The intra- and interday precision revealed coefficients of variation below 15%, and the accuracy was within the range of ±15%. The limits of quantification and detection obtained were 0.31 µg/mL for all compounds, except for DMT, where these values were 0.031 µg/mL [9]. When the analytical method was developed, the concentrations of the aforementioned compounds present in the ayahuasca decoctions were also determined. The concentrations of DMT ranged from 4.50 to 10.50 µg/mg extract (6.50 µg/mg in *P. viridis*, 10.50 µg/mg in *M. hostilis*, 10.40 µg/mg in the commercial mixture, 4.50 µg/mg in the mixture of *P. viridis* + *B. caapi*, 6.50 µg/mg in the mixture of *P. viridis* + *P. harmala*, 8.00 µg/mg in the mixture of *M. hostilis* + *B. caapi* and 8.50 µg/mg in the mixture of *M. hostilis* + *P. harmala*.

Concerning β-carbolines, harmine concentrations ranged between 0.02 and 12.00 µg/mg extract (10.00 µg/mg for *B. caapi*, 12.00 µg/mg for *P.harmala*, 0.02 µg/mg for the commercial mixture, 0.48 µg/mg for the mixture of *P. viridis* + *B. caapi*, 0.30 µg/mg for the mixture of *P. viridis* + *P. harmala*, 0.82 µg/mg for the mixture of *M. hostilis* + *B. caapi* and 9.00 µg/mg for the mixture of *M. hostilis* + *P. harmala*); harmaline concentrations ranged from 0.07 to 17.00 µg/mg extract (4.68 µg/mg for *B. caapi*, 17.00 µg/mg for *P. harmala*, 0.37 µg/mg for the commercial mixture, 0.07 µg/mg for the mixture of *P. viridis* + *B. caapi*, 0.48 µg/mg for the mixture of *P. viridis* + *P. harmala*, 0.12 µg/mg for the mixture of *M. hostilis* + *B. caapi* and 13.5 µg/ mg for the *M. hostilis* + *P. harmala* mixture).

For THH, the concentrations ranged between 0.63 and 5.00 µg/mg extract (5.00 µg/mg for *B. caapi*, 3.05 µg/mg for *P. harmala*, 2.09 µg/mg for the commercial mixture, 2.50 µg/mg for the mixture of *P. viridis* + *B. caapi*, 0.63 µg/mg for the mixture of *P. viridis* + *P. harmala*, 1.90 µg/mg for the mixture of *M. hostilis* + *B. caapi* and 3.44 µg/mg for the mixture of *M. hostilis* + *P. harmala*).

Harmol was also detected in extracts of samples of *B. caapi* (0.14 µg/mg), *P. harmala* (0.02 µg/mg), commercial mixture (0.01 µg/mg), *P. viridis* + *B. caapi* (0.01 µg/mg), *P. viridis* + *P. harmala* (0.02 µg/mg), *M. hostilis* + *B. caapi* (0.03 µg/mg), *M. hostilis* + *P. harmala* (0.06 µg/mg) and hamalol in the samples of *B. caapi* (0.05 µg/mg), *P. harmala* (0.66 µg/mg), *P. viridis* + *P. harmala* (0.08 µg/mg), *M. hostilis* + *B. caapi* (0.04 µg/ mg) and *M. hostilis* + *P. harmala* (0.36 µg/mg). These results are also presented in the study y Gonçalves et al. [9]. Regarding the aliquots collected in the present study when performing the PAMPA test, it was possible to verify that DMT, harmine, harmaline, THH, harmol and harmalol were not detected. To the best of our knowledge, PAMPA assays with ayahuasca have not been previously performed in NHDF cells. However, in a study carried out by our research team, where this same assay was performed in Caco2 cells, the presence of these compounds was verified [9]. It should be noted that in that work, an in vitro digestion was carried out, which may have facilitated the passage of the compounds [9]. Additionally, during that process, the DMT and β-carbolines present in the initial extracts were metabolised in compounds of smaller size, which more easily crossed the cell layer [9].

## 3. Materials and Methods

### 3.1. Chemicals and Materials

The analytical standards of DMT, harmine, THH, harmaline, harmalol and harmol were kindly provided by Nal von Minden, GmbH (Regensburg, Germany). Roswell Park Memorial Institute (RPMI) medium, 3-[4,5-dimethylthiazol-2-yl]-2,5-diphenyltetrazolium bromide (MTT) and Lucifer yellow were obtained from Sigma-Aldrich (Sintra, Portugal). Formic acid and dimethyl sulfoxide (99.9% of purity) were purchased from Sigma-Aldrich (Sintra, Portugal). Methanol (HPLC grade) was obtained from Fischer Chemical (Loughborough, UK). Deionised water was obtained from a Milli-Q System (Millipore, Billerica, MA, USA).

### 3.2. Sample and Work Solutions Preparation

Individual stock solutions of harmaline, harmol, harmalol, harmine and DMT were prepared at 1 mg/mL in methanol, and the working solutions were prepared from these by serial dilutions in methanol.

The vegetal samples were acquired online from Shayana Shop (https://www.shayanashop.com, Amsterdam, The Netherlands) (accessed on 25 May 2019). Ayahuasca decoctions were prepared according to a traditional recipe, provided by a consumer who was admitted to the emergency department with symptoms of intoxication. Thus, 0.210 g of each of the four plant samples—leaves of *P. viridis*, stem remains of *B. caapi*, root bark of *M. hostilis* and seeds of *P. harmala*—and the commercial mixture were weighed and then milled in a mortar with some water drops. After that, 250 mL of ultrapure water were added and the mixture was transferred to a flask and boiled for 4 h [2,9,27]. The four decoctions of the plant mixtures were prepared in the same way, with two of the above plant samples being mixed for each (*P. viridis* and *P. harmala*; *P. viridis* and *B. caapi*; *M. hostilis* and *P. harmala*; *M. hostilis* and *B. caapi*). After 4 h, the samples were cooled, filtered, frozen at –80 °C and lyophilised.

For application in cell culture, all lyophilised samples were prepared in RPMI medium at two concentrations (250 and 500 mg/L). These concentrations were used in other studies by our research group, in which antioxidant and anti-inflammatory properties were evaluated in other samples [2,18,20].

### 3.3. Cell Culture

The normal human dermal fibroblast (NHDF) cell line was cultured in RPMI medium supplemented with 0.01 M HEPES, 0.02 M L-glutamine, 0.001 M sodium pyruvate, 1% antibiotic mixture and 10% of foetal bovine serum, and then incubated at 37 °C in a humidified atmosphere containing 5% CO_2_. Cells were used between passages 23 and 28.

For the wound-healing and PAMPA assay, the cells were seeded in culture inserts placed in 12 multi-well plates (at a cell density of 6 × 10^4^). On the other hand, for the MTT assay, the cells were seeded in 96 multi-well plates at a cell density of 0.5 × 10^4^.

#### 3.3.1. Cytotoxicity

The cytotoxicity of the samples was evaluated by the cell viability assay using the MTT method. Thus, after the cells reached confluence in 96-well plates, they were exposed to the samples for 24 h. The negative control consisted of RPMI medium. After the incubation time, the medium from all the wells was removed and replaced with an MTT solution (0.5 mg/mL). Then, the cells were incubated at 37 °C for 3 h. After that, the MTT solution was removed and the formazan crystals were dissolved in dimethylsulfoxide (DMSO), the absorbance being measured at 570 nm in a microplate reader. All the assays were performed in triplicate.

#### 3.3.2. Wound-Healing Assay

The assay was carried out in accordance with previously developed studies [18,19,20]. For this purpose, the selected samples were submitted to the wound scratch assay to assess their healing activity. Thus, after the NHDF cells reached confluence, the RPMI medium was removed, and a scratch was created by scraping in a straight line with a p200 micropipette tip. Then, reference points were marked on the microplate, so that the same point was always visualised. The wells were washed with PBS and after that, samples were added to the cells, using RPMI medium as a negative control. After that, the plates were placed under a phase contrast microscope and images were acquired at the initial time (t = 0 h). Then the cells were incubated at 37 °C with the samples and new images were taken after 2, 8, 12 and 24 h. The distances between the lesion margins were evaluated using a digital image analysis tool (IC Measure software version 2.0.0.161, The Imaging Source, Bremen, Germany). All assays were performed in triplicate.

#### 3.3.3. Parallel Artificial Membrane Permeability Assay

The potential crossing of psychoactive compounds by NHDF cells was evaluated using the PAMPA assay. Thus, after a confluent monolayer was formed, the samples were placed in contact with it by adding 500 μL of each sample to the apical chamber. The cells were incubated at 37 °C and after 2, 8, 12 and 24 h aliquots of 250 μL were collected from the basolateral chamber. Subsequently, the collected aliquots were analysed by high performance liquid chromatography-diode array detector (HPLC-DAD). All assays were performed in triplicate.

#### 3.3.4. Transepithelial Electrical Resistance Assay

Cell monolayer integrity, as well as tight junction changes, were assessed by measuring transepithelial electrical resistance (TEER). Thus, before and after the PAMPA assay, TEER was measured using a transepithelial resistance meter electrode (EVOM2, World Precision Instrument, Sarasora, FL, USA). Thus, the electrode was equilibrated with culture medium and then placed in each well, making a 90° angle. The longest part remained in the basolateral chamber, while the shortest part remained in the apical chamber. The assay was performed in triplicate and the TEER value was determined according to the following equation:

TEER value = (mean of the resistances of each well-mean of the resistance of blank) × insert area (1)

#### 3.3.5. Lucifer Yellow Permeability Assay

Cell monolayer permeability changes were evaluated by the Lucifer yellow permeability assay. Thus, after performing the PAMPA assay, the medium of the apical and basolateral compartments was removed and replaced with 500 µL of Lucifer yellow solution (0.1 mg/mL) in the apical chamber and 1.5 mL of Hank’s balanced salt solution (HBSS) in the basolateral chamber. Cells were incubated at 37 °C for 1 h. After this, 200 µL were collected from each basolateral chamber and transferred to a 96-well plate. A Lucifer yellow (0.1 mg/mL) solution was used as a positive control and HBSS buffer was used as a blank. Then fluorescence was measured at 485 nm (excitation) and 535 nm (emission) using a spectrofluorometer. The permeability percentage was calculated as follows:

% permeability = (mean of fluorescence of each well-fluorescence of blank)/(fluorescence of positive control-fluorescence of blank) × 100 (2)

### 3.4. Instrumental and Chromatographic Conditions

The quantification of DMT, harmine, harmaline, THH, harmol and harmalol was performed in an HPLC-DAD (Agilent Technologies, Soquímica, Lisbon, Portugal). The injection volume was 50 µL and the flow rate was 1.5 mL/min. The stationary phase consisted of a YMC-Triart PFP analytical column (5 m, 4.6 i.d. × 150 mm) coupled to a Guard-c support (4 × 10 mm) and a Triart PFP pre-column (5 m, 3 × 10 mm), all from YMC Europe GMBH (Solítica, Lisbon, Portugal). The oven temperature was maintained at 25 °C. The mobile phase consisted of 0.1% formic acid in methanol in line A, and 0.1% formic acid in water in line B. Elution was performed in gradient mode and included 5% A (0–2 min), 50% A (2–32 min) and again, 5% A (32–40 min). Finally, harmaline and harmalol were detected at 360 nm, DMT and THH at 278 nm and harmine and harmol at 246 nm. The sampler temperature was set at 4 °C.

### 3.5. Statistical Analysis

The results were expressed as mean values with standard deviations (SD). The Student’s *t*-test was employed, and statistically significant values were considered when *p* < 0.05 (*).

## 4. Conclusions

Ayahuasca samples showed a great healing potential, which can be seen in the microscopic images collected. After performing the PAMPA assay with the extracts, it was also possible to verify that they did not show cytotoxicity, and the integrity of the cell monolayer remained unchanged, except for one sample, as well as its permeability. This suggests that most samples did not interfere with intercellular junctions. It should be noted that the present study is the first work where these tests were carried out simultaneously, and additionally, it was an in vitro test; therefore, conclusions must be drawn with caution.

## Figures and Tables

**Table 1:** Cell viability after exposure to extracts. The values are expressed as mean ± SD.

Samples	Cell Viability (%)	
	250 mg/L	500 mg/L
*P. viridis*	150.24 ± 0.18	130.22 ± 0.08
*B. caapi*	91.72 ± 0.17	134.80 ± 0.09
*P. harmala*	38.07 ± 0.03	24.91 ± 0.001
*M. hostilis*	95.38 ± 0.11	121.79 ± 0.00
Commercial mixture	77.86 ± 0.11	98.35 ± 0.01
*P. viridis* + *B. caapi*	110.25 ± 0.08	229.85 ± 0.07
*P. viridis* + *P. harmala*	74.11 ± 0.06	174.54 ± 0.02
*M. hostilis* + *B. caapi*	111.89 ± 0.12	160.62 ± 0.03
*M. hostilis* + *P. harmala*	136.19 ± 0.03	132.42 ± 0.09

**Table 2:** Microscopic images obtained from the scratch wound-healing assay with the samples of ayahuasca (magnification: 100×). The margins of the scratch appear in red.

Representative Image of the Cells at the Initial Moment (0 h)				
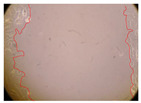				
	**2 h**	**8 h**	**12 h**	**24 h**
Control	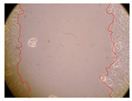	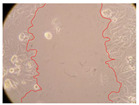	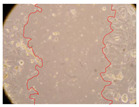	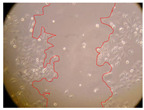
*P. viridis* 250 mg/L	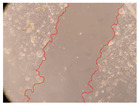	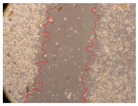	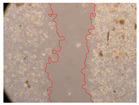	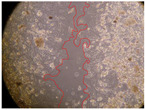
*P. viridis* 500 mg/L	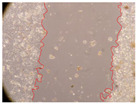	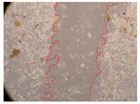	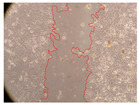	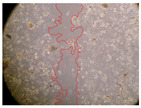
*B. caapi* 250 mg/L	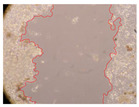	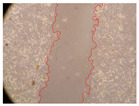	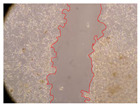	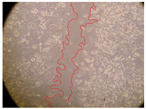
*B. caapi* 500 mg/L	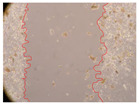	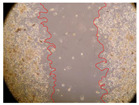	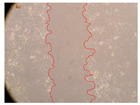	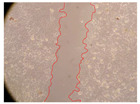
*M. hostilis* 250 mg/L	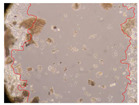	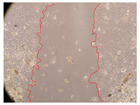	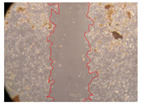	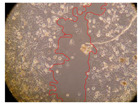
*M. hostilis* 500 mg/L	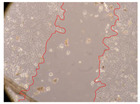	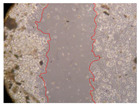	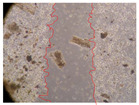	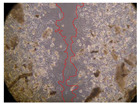
Commercial mixture 250 mg/L	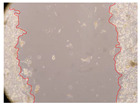	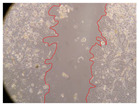	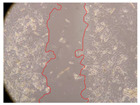	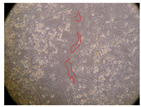
Commercial mixture 500 mg/L	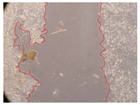	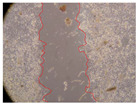	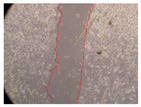	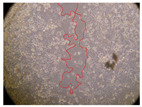
*P. viridis* + *B. caapi* 250 mg/L	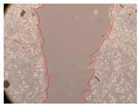	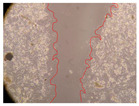	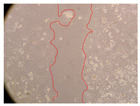	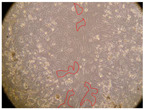
*P. viridis* + *B. caapi* 500 mg/L	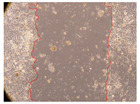	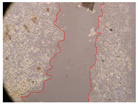	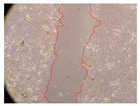	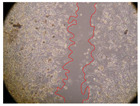
*P. viridis* + *P. harmala* 250 mg/L	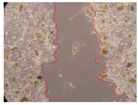	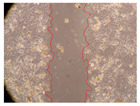	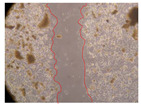	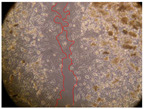
*P. viridis* + *P. harmala* 500 mg/L	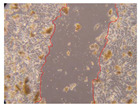	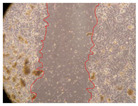	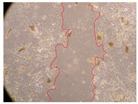	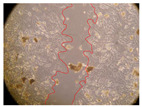
*M. hostilis* + *B. caapi* 250 mg/L	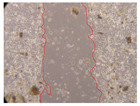	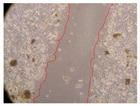	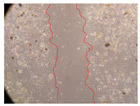	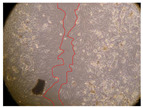
*M. hostilis* + *B. caapi* 500 mg/L	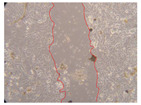	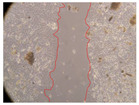	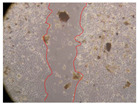	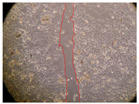
*M. hostilis* + *P. harmala* 250 mg/L	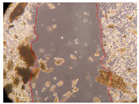	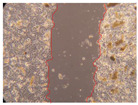	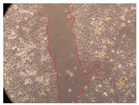	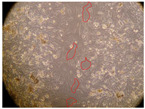
*M. hostilis* + *P. harmala* 500 mg/L	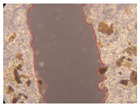	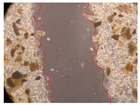	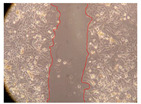	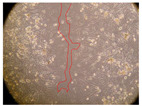

**Table 3:** Calculated mean difference between the distance of the injury of the negative control and the samples.

Samples	0 h	2 h	*p*-Value	8 h	*p*-Value	12 h	*p*-Value	24 h	*p*-Value
Control	3.8	3.79	-	3.13	-	2.93	-	2.65	-
*P. viridis* 250 mg/L		2.36	<0.001	2.06	<0.001	1.5	<0.001	0.53	<0.001
*P. viridis* 500 mg/L		2.97	<0.001	2	<0.001	1.64	<0.001	0.54	<0.001
*B. caapi* 250 mg/L		3.23	<0.001	1.71	<0.001	1.42	<0.001	0.75	<0.001
*B. caapi* 500 mg/L		3.08	<0.001	2.24	<0.001	1.42	<0.001	1	<0.001
*M. hostilis* 250 mg/L		4.14	<0.001	2.43	<0.001	1.49	<0.001	0.81	<0.001
*M. hostilis* 500 mg/L		2.43	<0.001	2.07	<0.001	1.76	<0.001	0.4	<0.001
Commercial mixture 250 mg/L		3.79	1	2.05	<0.001	1.39	<0.001	0.49	<0.001
Commercial mixture 500 mg/L		3.14	<0.001	1.85	<0.001	1.11	<0.001	0.69	<0.001
*P. viridis* + *B. caapi* 250 mg/L		2.10	0.001	1.38	<0.001	1.23	<0.001	0.25	<0.001
*P. viridis* + *B. caapi* 500 mg/L		2.73	<0.001	1.5	<0.001	1.29	<0.001	0.63	<0.001
*P. viridis* + *P. harmala* 250 mg/mL		1.95	<0.001	1.44	<0.001	1.26	<0.001	0.37	<0.001
*P. viridis* + *P. harmala* 500 mg/L		2.15	<0.001	1.98	<0.001	1.34	<0.001	0.8	<0.001
*M. hostilis* + *B. caapi* 250 mg/L		2.08	<0.001	1.65	<0.001	1.38	<0.001	0.47	<0.001
*M. hostilis* + *B. caapi* 500 mg/L		1.72	<0.001	1.38	<0.001	1.2	<0.001	0.33	<0.001
*M. hostilis* + *P. harmala* 250 mg/L		2.83	<0.001	1.71	<0.001	1.07	<0.001	0.3	<0.001
*M. hostilis* + *P. harmala* 500 mg/L		2.73	<0.001	2	<0.001	1.47	<0.001	0.2	<0.001

**Table 4:** TEER values obtained before and after incubation with the extracts. The values are expressed as mean ± SD. Statistically significant values were considered if *p* < 0.05.

Samples	TEER (Ω cm^2^)		
	Before	After	*p*-Value
Control	924 ± 124.45	1166 ± 217.79	0.306
*P. viridis* 250 mg/L	1166 ± 31.11	1056 ± 62.23	0.155
*P. viridis* 500 mg/L	1122 ± 155.56	1188 ± 248.90	0.781
*B. caapi* 250 mg/L	1309 ± 140.01	1100 ± 124.45	0.255
*B. caapi* 500 mg/L	1023 ± 202.23	1430 ± 93.34	0.123
*M. hostilis* 250 mg/L	1342 ± 93.34	1320 ± 124.45	0.860
*M. hostilis* 500 mg/L	869 ± 171.12	968 ± 186.68	0.636
Commercial mixture 250 mg/L	1265 ± 233.35	1056 ± 0.00	0.333
Commercial mixture 500 mg/L	836 ± 62.23	1144 ± 124.45	0.089
*P. viridis* + *B. caapi* 250 mg/L	858 ± 31.11	1254 ± 217.79	0.126
*P. viridis* + *B. caapi* 500 mg/L	1078 ± 217.79	1386 ± 31.11	0.186
*P. viridis* + *P. harmala* 250 mg/L	1254 ± 31.11	1298 ± 31.11	0.293
*P. viridis* + *P. harmala* 500 mg/L	1056 ± 62.23	1298 ± 31.11	0.039
*M. hostilis* + *B. caapi* 250 mg/L	979 ± 202.23	1342 ± 155.56	0.182
*M. hostilis* + *B. caapi* 500 mg/L	1331 ± 202.23	1276 ± 124.45	0.774
*M. hostilis* + *P. harmala* 250 mg/L	924 ± 186.68	1056 ± 0.00	0.403
*M. hostilis* + *P. harmala* 500 mg/L	968 ± 124.45	1210 ± 155.56	0.228

**Table 5:** Percentage of permeability of NHDF cells after incubation with the extracts. The values are expressed as mean ± SD. Statistically significant values were considered if *p* < 0.05.

Samples	Permeability (%)	*p*-Value
Control	13.55 ± 0.51	-
*P. viridis* 250 mg/L	14.69 ± 3.04	0.652
*P. viridis* 500 mg/L	13.75 ± 2.49	0.922
*B. caapi* 250 mg/L	13.40 ± 2.65	0.947
*B. caapi* 500 mg/L	13.41 ± 2.76	0.951
*M. hostilis* 250 mg/L	15.46 ± 2.98	0.465
*M. hostilis* 500 mg/L	14.40 ± 2.74	0.709
Commercial mixture 250 mg/L	13.80 ± 2.74	0.910
Commercial mixture 500 mg/L	12.33 ± 0.73	0.193
*P. viridis* + *B. caapi* 250 mg/L	12.69 ± 0.29	0.174
*P. viridis* + *B. caapi* 500 mg/L	12.51 ± 2.46	0.617
*P. viridis* + *P. harmala* 250 mg/L	11.33 ± 1.29	0.151
*P. viridis* + *P. harmala* 500 mg/L	11.29 ± 0.38	0.037
*M. hostilis* + *B. caapi* 250 mg/L	12.51 ± 2.55	0.628
*M. hostilis* + *B. caapi* 500 mg/L	13.66 ± 0.05	0.789
*M. hostilis* + *P. harmala* 250 mg/L	14.52 ± 1.45	0.467
*M. hostilis* + *P. harmala* 500 mg/L	13.85 ± 0.12	0.501

## Data Availability

Not applicable.

## Sample Availability

Samples of the compounds are available from the authors.
